# Toward mapping pragmatic impairment of autism spectrum disorder individuals through the development of a corpus of spoken Japanese

**DOI:** 10.1371/journal.pone.0264204

**Published:** 2022-02-25

**Authors:** Sumi Kato, Kazuaki Hanawa, Vo Phuong Linh, Manabu Saito, Ryuichi Iimura, Kentaro Inui, Kazuhiko Nakamura

**Affiliations:** 1 Department of Neuropsychiatry, Graduate School of Medicine, Hirosaki University, Hirosaki, Japan; 2 Faculty of Management and Law, Aomori Chuo Gakuin University, Aomori, Japan; 3 Natural Language Understanding Team, RIKEN Center for Advanced Intelligence Project, Tokyo, Japan; 4 Natural Language Processing Lab, Graduate School of Information Sciences, Tohoku University, Sendai, Japan; 5 School of Global Studies, University of Sussex, Brighton, United Kingdom; 6 Research Center for Child Mental Development, Graduate School of Medicine, Hirosaki University, Hirosaki, Japan; 7 Department of International Management, College of Business Administration, Tamagawa University, Tokyo, Japan; Kanazawa University Graduate School of Medical Sciences: Kanazawa Daigaku Daigakuin Iyaku Hokengaku Sogo Kenkyuka Iyaku Hoken Gakuiki Igakurui, JAPAN

## Abstract

The central symptom of autism spectrum disorder (ASD) is deficiency in social communication, which is generally viewed as being caused by pragmatic impairment (PI). PI is difficulty in using language appropriately in social situations. Studies have confirmed that PI is the result of neurological, cognitive, linguistic, and sensorimotor dysfunctions involving intricately intertwined factors. To elucidate the whole picture of this impairment, an approach from a multifaceted perspective fusing those factors is necessary. To this end, comprehensive PI mapping is a must, since no comprehensive mapping has yet been developed. The aim of this research is to present a model of annotation scheme development and corpus construction to efficiently visualize and quantify for statistical investigation occurrences of PI, which enables comprehensive mapping of PI in the spoken language of Japanese ASD individuals. We constructed system networks (lexicogrammatical option systems speakers make choices from) in the theoretical framework of Systemic Functional Linguistics, from which we developed an annotation scheme to comprehensively cover PI. Since system network covers all possible lexicogrammatical choices in linguistic interaction, it enables a comprehensive view of where and in what lexicogrammar PI occurs. Based on this annotation scheme, we successfully developed the *Corpus of ASD + Typically Developed Spoken Language* consisting of texts from 1,187 audiotaped tasks performed by 186 ASD and 106 typically developed subjects, accommodating approximately 1.07 million morphemes. Moreover, we were successful in the automatization of the annotation process by machine learning, accomplishing a 90 percent precision rate. We exemplified the mapping procedure with a focus on the spoken use of negotiating particles. Our model corpus is applicable to any language by incorporating our method of constructing the annotation scheme, and would give impetus to defining PI from a cross-linguistic point of view, which is needed because PI of ASD reflects cross-linguistic differences.

## Introduction

### Background

Autism spectrum disorder (ASD) is a neurodevelopmental disorder characterized by persistent deficits in social communication and social interaction across multiple contexts, as well as by restricted, repetitive patterns of behavior, interests, or activities [1]. The core symptom is deficiency in social communication which is caused by pragmatic impairment (PI) [2, 3]. The definition of PI is difficulty in language comprehension and production with regards to using language appropriately in social settings. Pragmatics refers to the socially customary way to use and understand language in social settings, including changing language according to the situation (e.g. formal/informal), the use and understanding of implied meanings or non-literal language (e.g. idiom, metaphor, irony, sarcasm), and the contribution of relevant, informative utterances to an interpersonal interaction. Deficits in the pragmatic use of language invite barriers to effective communication and the construction of interpersonal relationships.

Traditionally, pragmatics belongs to the fields of philosophy and linguistics [4], where its theoretical framework is perceived as centered solely on language and its discussion is completed within that frame [5–8]. On the other hand, clinicians in urgent need of appropriate interventions for pragmatic disorders have developed their own approaches [4]. Studies of PI in the clinical field have dealt with nonverbal aspects of communication, such as gaze, gesture, posture, and social rapport, rather than focusing on language [4]. For example, Dronker et al., in their investigation of severe Broca’s aphasia and deafness, found that nonverbal social skills and abilities existed in parallel with linguistic abilities, both of which could be treated as independent phenomena [9]. PI is not an ASD-specific phenomenon but is also seen in a wide range of disorders, such as aphasia, right hemisphere damage, cognitive impairment such as Alzheimer’s disease, and traumatic brain injury [10–12].

Meanwhile, clinicians have long been aware of ASD individuals who had reasonably good language skills but could not communicate well. These facts have led them to understand that cognitive functions such as inferential reasoning, executive function, and memory play an important role in interpersonal interaction, from which it has been argued in the clinical field that cognition is closely associated with PI [4]. From this understanding, neurology-based research, especially, has become a major focus of studies of PI [13].

**Fig 1:**
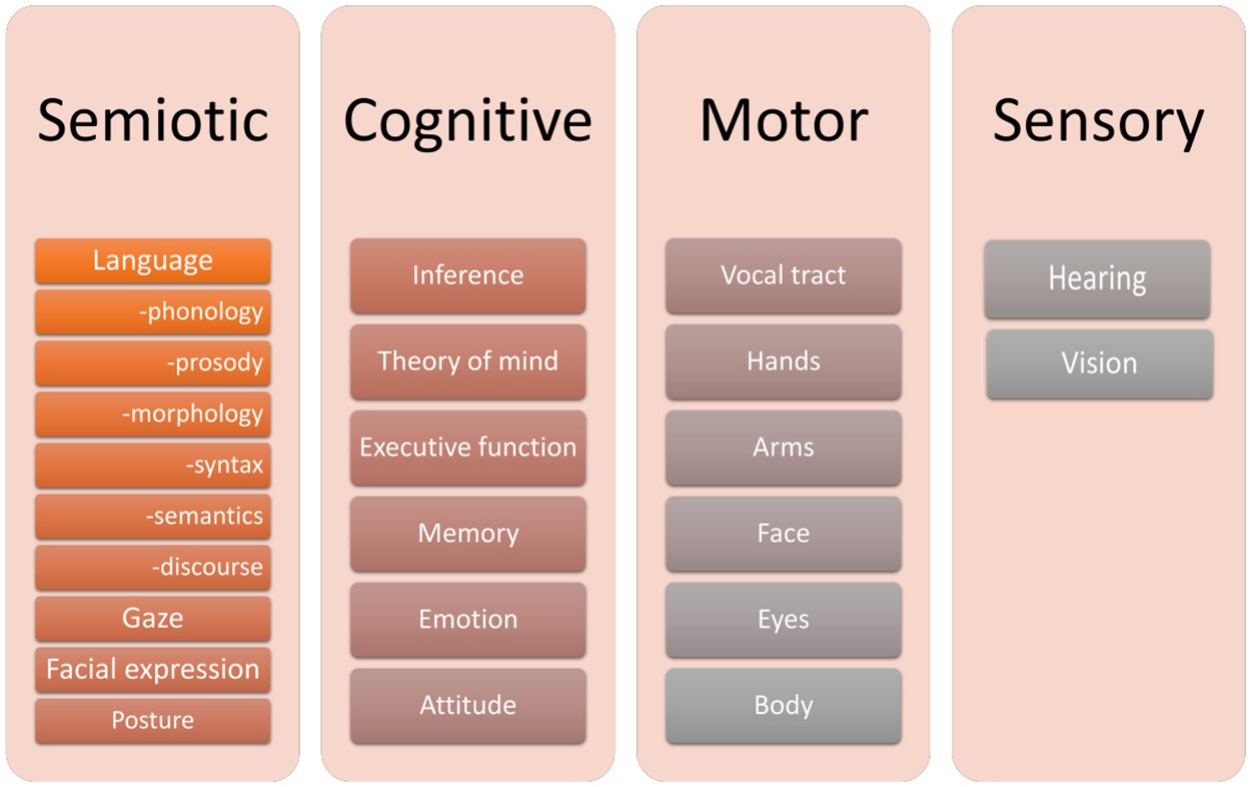
Elements of pragmatics (adapted from Perkins [4]).

In view of the above, there has been a widespread recognition among researchers in the clinical field that PI should be considered more comprehensively by combining multiple factors such as language, nonverbal aspects, and cognition. Many previous studies have argued that PI is the result of neurological, cognitive, symbolic, and sensorimotor dysfunction [4, 14–17]. Perkins lists four areas ― semiotic, cognitive, motor, and sensory ― as elements of pragmatics, as shown in Fig 1 [4].

**Fig 2:**
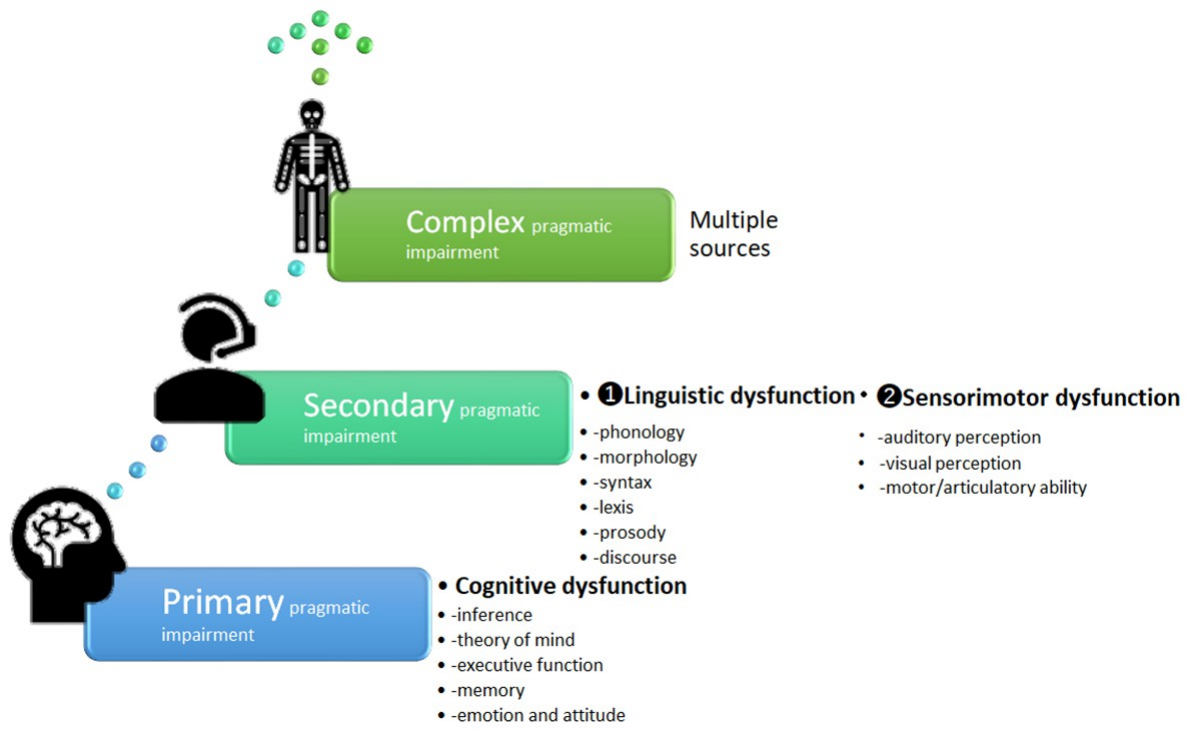
A classification scheme for PI and underlying causes (adapted from [4]).

Furthermore, in reference to PI, Perkins proposed a classification scheme prioritizing these factors: cognitive dysfunction as primary and linguistic and sensorimotor dysfunction as secondary PI, as shown in Fig 2 [4].

### Research in the domain of language

Among studies that investigate concrete linguistic phenomena of ASD, there are those dealing with a single lexicogrammatical area such as modality [18–23]. Perkins [18, 19] along with Nuyts and Roeck [20], reported limited understanding and use of epistemic modal expressions by ASD children from their story-narrating experiments. Tager-Flusberg found that there are different usages of modal expressions, especially of *can* and *will*, between ASD children and Down syndrome children [23]. In these studies, the observed linguistic phenomena are collated with explanations from the perspective of cognitive dysfunction. The problem is that these investigations are limited to a specific lexicogrammatical area; the whole picture of the impairment has not been systematized. Comprehensive mapping that systematically identifies the linguistic phenomena and instances of pragmatic disorder will be needed to elucidate PI as a whole. However, no comprehensive and systematic mapping of PI in the domain of linguistics has been made so far.

One of the reasons why comprehensive linguistic research is needed is that a supplementary diagnostic tool—one that helps to identify ASD by language usage—would be highly desirable. Previous studies have simply listed instances of PI with a view to clinical application. Instances of PI are evidenced in diverse linguistic domains. Penn points out response to interlocutor, control of semantic content, cohesion, fluency, sociolinguistic sensitivity, and nonverbal communication [24]. Prutting and Kirchner list 18 pragmatic verbal behaviors including speech acts, topic, turn taking, lexical selection, accuracy, cohesion, and stylistic variations, together with paralinguistic aspects and nonverbal aspects [25]. Bishop also presents a similar checklist referring to speech output, including intelligibility and fluency, syntax, inappropriate initiation, coherence, stereotyped conversation, use of conversational context, conversational rapport, social relationships, and interests [26]. In Prutting and Kirchner’s and Bishop’s checklists, as far as the verbal aspect is concerned, it seems that the checked items are rather abstract; as a result, the evaluation tends not to be immune from arbitrariness. This makes it difficult to maintain consistency among raters. Thus the clinical implementation of their system is not realistic, since it entails a quite complicated procedure [27]. A simpler evaluation tool that does not require intricate procedures is needed in order to efficiently promote clinical practice based on the overall picture of PI [27].

### Related existing corpora

One of the most effective ways to comprehensively map PI of ASD is by use of corpora of the spoken language of ASD individuals. Although research based on this is scarce, among those for English is Parish-Morris’s, which is not open to the public [28]. It consists of a pilot corpus and an extension corpus which were built from an audio-recorded version of the ADOS (Autism Diagnostic Observation Schedule) administration. Through this corpus, differences in speaking rate and inter-turn gaps between ASD and typically developed (TD) individuals have been observed. Parish-Morris’s is a work in progress, with plans to increase annotation. Among the open corpora is the *Nadig ASD English Corpus*, which is a collection of transcripts of videotaped free play between ASD children (n = 38: 36–74 months) and their parents [29]. It is a raw collection of simple linguistic data with no semantic information annotated. Similarly, *Asymmetries ASD Corpus* is a corpus of Dutch-speaking ASD and TD individuals’ spoken language (n = 192: child-elderly), which is also raw in form [30]. To the best of our knowledge, Sakishita et al. is the only previous study of Japanese-speaking ASD individuals’ spoken language using an originally constructed corpus [31]. Sakishita’s version has 17 kinds of annotation based on the publicly available *Chiba 3 Party* (this is a chat of 12 pairs of friends recorded at Chiba University). Sakishita’s *Annotation Criteria*: pause, stretch, clog, word break, upswing, filler, response interjection, suspended clause, misrepresentation, words that cannot be written in kanji, Chinese characters, songs, inaudible speech sounds, laughter, breathing, reading kana, and Japanese syllables. Using these annotations and morpheme information, statistics are derived and the correlation with the ADOS scores is investigated. The difference from our corpus is that ours specializes in the lexicogrammar used by individuals with ASD, while Sakishita’s concentrates on their phonetic usage.

We are not aware of any previous study that has presented a corpus entailing comprehensive annotation of the lexicogrammar of ASD individuals’ spoken language. The corpus we constructed for this study annotates syntax and lexis since PI in ASD appears in skewed lexical choices, which are identified as lexical anomalies [32, 33]. In ASD individuals, the lexical processing problem is the most frequently cited example of PI related to semantic choice [32, 33].

### Objective

The objective of this research is to create a model corpus providing a mechanism to effectively and efficiently visualize and quantify, for statistical investigation, where PI occurs and in what linguistic expressions it manifests in the spoken language of ASD individuals. This is to elucidate the linguistic dysfunction, which is the secondary PI illustrated in Fig 2, with a particular focus on syntax and lexis. To this end, we constructed system networks (lexicogrammatical option systems which speakers make choices from) in the theoretical framework of Systemic Functional Linguistics (SFL), from which we developed an annotation scheme to cover PI from a comprehensive view of the Japanese language. Furthermore, through machine learning, we aimed to automatize annotation with a 90 percent precision rate, which allows for significant reductions in cost, time, and manpower, facilitating the extension and growth in size of the corpus as a monitor corpus to make the findings more reliable, since constructing a diagnostic differentiation tool entails enormous amounts of these resources.

A basic assumption of this research is that the lexicogrammatical choices of individuals with ASD which differ significantly from those of TD individuals should be regarded as evidence of PI. This is because utterances made by TD individuals can be regarded as majority speech in line with social practices, and utterances that deviate from majority speech can be regarded as instances of PI. This study assumes that spoken items that evidence a significant difference from majority speech differ diagnostically between ASD and TD. We hypothesized model flow of PI.

Based on this assumption, we can inductively reason which cognitive function is deficient or impaired by clarifying the tendency of individuals with ASD to use or not use a certain lexicogrammar properly. Since each item of lexicogrammar has its own linguistic function, collating those instances of inappropriate use or non-use of specific lexicogrammar inductively leads us to understand which area of cognition is impaired or deficient. Negotiating particles are among the items of PI lexicogrammar that we have so far identified in this study. We exemplified the procedure of judging that the use or non-use of negotiating particles represents pragmatic impairment in ASD individuals.

## Method

### (1) Sampling

#### Participants

The participants were individuals with ASD, along with TD individuals. The ASD participants were divided into two groups. One group (ASD-1; N = 56) consisted of subjects who had been clinically diagnosed based on DSM-5 [1]. The other ASD group (ASD-2; N = 130) were subjects diagnosed based on DSM-5 while using the Autism Diagnostic Observation Schedule (ADOS-2) as a diagnostic aid. ADOS is highly evaluated as the gold standard demonstrating strong predictive validity. Measurement is based on observation and interaction, with the individual suspected of having ASD being assessed for reciprocal social interaction, communication, and imagination in a semi-structured setting. ADOS-2 was conducted by the administrator who established research reliability required for research use of the results of ADOS administration. Coding the observed behavior through scoring algorithms results in diagnostic measurement of the autism symptoms. Scores are compared using an algorithm cut-off score for ASD. If the subject’s score meets or exceeds cut-offs in all three areas, reciprocal social interaction, communication, and restricted and repetitive behaviors, they are considered to meet the criteria for that classification.

The TD group (N = 106) were subjects who did not meet the psychiatric diagnosis. Table 1 shows subject information and the number of samples included in the corpus.

**Table 1:** Subject group information.

subject group	number of subjects	age range	age mean	age standard deviation	number of samples
ASD-1	56	8–38	12.6	7.3	192
ASD-2	130	3–46			531
TD	106	3–31	13.8	6.9	464
Total	292				1187

#### Tasks

The spoken language was audiotaped from the six to eight tasks given to the participants with potential diagnosis of ASD during the ADOS-2 administration. In addition, the tasks performed included those separate from ADOS-2 administration. Table 2 shows the task descriptions. Tasks A, E, DG, P, CT, SM, G are from the ADOS administration. The rest of the tasks were decided on considering that they were well-structured to elicit clinically significant indications of behaviors directly related to a diagnosis of ASD.

**Table 2:** Task description.

Task ID	Remarks
A	Interview
E	Narrative of a picture book without words ^a^
D	Description of photographs ^b^
DG	Demonstration tasks from ADOS-2 administration; demonstration and reporting of toothbrushing
P	Description of a picture from ADOS-2 administration ^c^
CT	Narrative of a cartoon story from ADOS-2 administration ^d^
SM	Creation of a story using five objects either with a definite purpose or with no clear purpose from ADOS-2 administration ^e^
H	Role-play dialog
I	Description of pictures from a picture book ^f^ ^g^
B	Description of the emotion represented in the pictures ^h^
C	Description of photographs ^i^
G	Creation of a story using a situation-setting sheet and cartoon characters ^j^

^a^ David Wiesner. Tuesday. 1991. Houghton Mifflin Company.

^b^ What Are They Thinking? ColorCards. Creative Therapy Store.

^c^ ADOS-2: Description of a Picture Task: Picture Card. 1999, 2012. Western Psychological Services.

^d^ ADOS-2: Cartoon Task: Series A/Card 6. 1999, 2012. Western Psychological Services.

^e^ ADOS-2: Creating a Story Task: 1999, 2012. Western Psychological Services.

^f^ Anno, Mitsumasa. 1978. Tabi no ehon [Travel picture book 2]. Tokyo: Fukuinkan Shoten Publisher Inc.

^g^ Anno, Mitsumasa. 1968. Fushigina e [strange pictures]. Tokyo: Fukuinkan Shoten Publisher Inc.

^h^ Feeleez. Creative Therapy Store.

^i^ A Box Full of Feelings. Creative Therapy Store.

^j^ The Story Telling Card Game (by Richard A. Gardner, MD). Creative Therapy Store.

#### Transcribing

Audiotaped tasks were transcribed by six college-educated native speakers of Japanese (junior transcribers). After that, three other college-educated native speakers of Japanese (senior transcribers) performed a check of the transcripts according to transcription specifications.

#### Ethics statement

The research was performed in accordance with the ethical guidelines of the Declaration of Helsinki. The protocol of this study was approved by the Committee on Medical Ethics of Hirosaki University (IRB 2013–142, 2018–168). To protect personal data, we adhered to the committee’s information security policies. Written consent was obtained for subjects aged 20 and older, and parent/guardian written consent as well as subject consent was obtained for subjects aged 19 and younger. Personal privacy was protected by using alphanumeric characters for identifiers of the subjects and proper nouns related to subjects. In addition, utterances that could identify specific individuals were deleted from the transcripts.

### (2) Constructing the system network (the choice system) in the Japanese language

The tagged information of semantic analysis is based on the system network of SFL elaborated by Halliday [34]. SFL views linguistic activity as a stratified system related to social context. Social context in this case means all the combined social constituent factors such as social system, culture, and consuetude.

**Fig 3:**
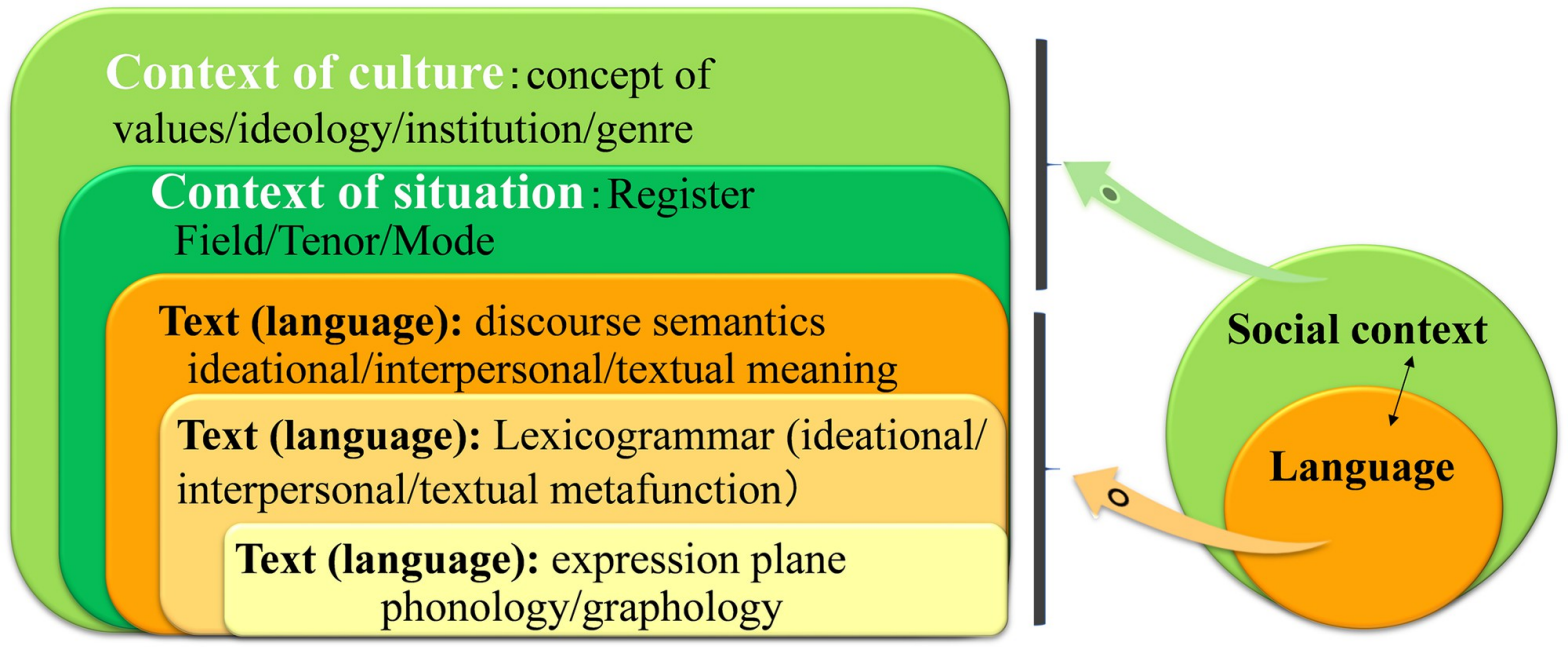
Bidirectional relationship between language and social context and the stratification of language. This is based on Martin [35].

Fig 3 illustrates the systemic model of language strata constructed in SFL and the interconnection between the stratified layers of language activity. In this model, the uppermost stratum is the context of culture, which represents the aggregation of sociocultural factors (such as social value and ideology) specific to the society in which a given language is used. Social activity is firstly defined by context of culture in making choices of lexicogrammatical resources. The second layer is context of situation, which concerns register. All interpersonal interaction is established by the choice of meaning, which is confined to the specific range these two contexts cover.

The circles on the right side in Fig 3, which are a simplification of the left side of the diagram, illustrate the bidirectional relationship between the social context and language, in that “language serves as the realization of and expression plane for social context, and in turn, social context is the content plane for language and is a more abstract level of sociosemantic representation” [36]. The ranks on the left side of the diagram form a stratified version of the right-side circles, illustrating how a communicator deploys meaning potential in an actual meaning exchange. Register reflects on its down-ranking strata, including lexicogrammar, by influencing the choices which speakers make to create meaning. In particular, field (what is going on, what the topic under discussion is) subsumes ideational meanings, tenor (the social and contextual roles of interactants) subsumes interpersonal meanings, and mode (the communication mode the interaction takes) subsumes textual meanings [36]. Further down the ranks, discourse semantics is realized by lexicogrammar, and lexicogrammar by phonology/graphology. From these interconnections between context and language, a communicator is capable of inferring a context from a given instance of language and presaging language patterns from a given situation. Accurate perception and full understanding of both cultural and situational contexts enable a speaker to make appropriate use of the lexicogrammar; otherwise, odd lexicogrammatical choices are made and PI occurs.

One of the central organizing concepts in SFL is language choice. We use different expressions depending on the person we are talking to, the scene, and various other factors. Thus, when constructing a certain clause for representing what we want to mean, there are some options, and the speaker instantly makes choices from the resource-selection mapping for each part of the clause at the time of utterance. SFL calls this resource-selection mapping the system network, which covers all the possible lexicogrammar for the speaker to choose in linguistic interaction. Language is a meaning-making system in which speakers have choice in their selection of resources from the system network when they engage in social activity [37]. In other words, to delineate all lexicogrammatical choices comprehensively is to fit the description of a language system which SFL aims at. Thus, building an annotation scheme based on this system network denotes building it from the perspective of the entire language, which makes the current annotation scheme a comprehensive one.

**Fig 4:**
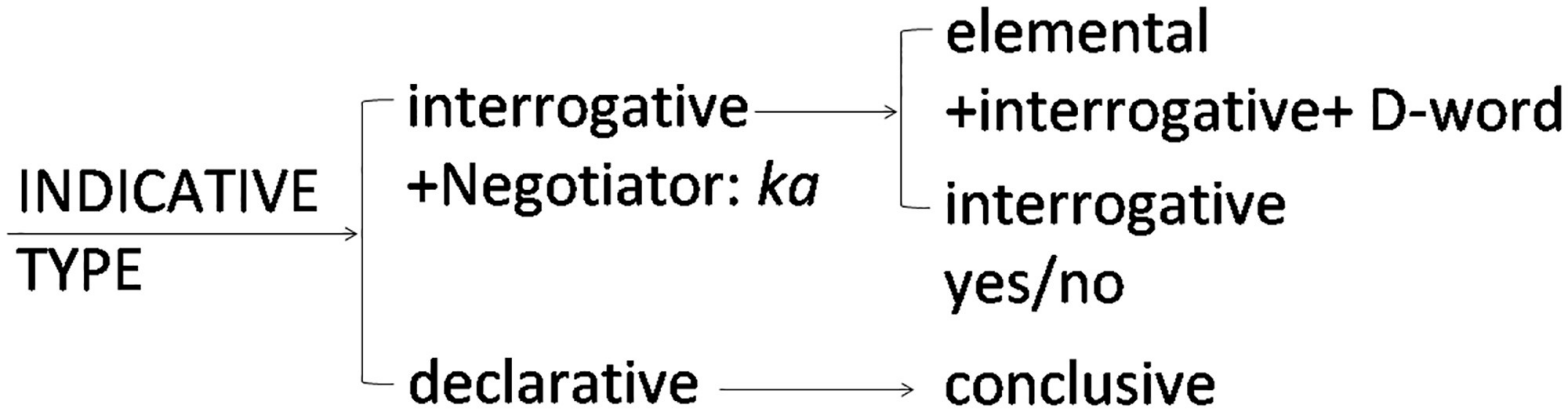
Indicative type, from the system network circled in red (1) in Fig 7.

Systems in SFL are construed as networks of paradigmatic oppositions. Paradigmatic systems are ways of representing the meaning-making potential of language constructs (i.e., the options of language constructs from which a speaker may choose). We will take a simple example of the therapist’s utterance in a therapeutic interview, “What makes you feel anxious or uneasy?” We will analyze this sentence in the system network of mood selection shown in Fig 4, which is an enlargement of the red-circled Part 1 in the system of MOOD in Fig 7, which we constructed for this study. The degree of delicacy increases going from left to right on the network. If the speaker chooses an interrogative approach rather than declarative, as in this question, then the next choice is opened up between polar or d-word interrogatives (typically staring with /d/, e.g., *dore* (which) and *dare* (who), which corresponds to wh- question words in English). Thus system networks provide speakers with choices that enable all the different kinds of grammatical realizations possible.

The annotation scheme of the current corpus was made based on the Japanese system network constructed for this project, using the method of transfer comparison. System network is the description of a particular language which brings out the special features of that language [38]. The approach of describing a particular language with no assumptions being made based upon other languages takes an inordinate amount of time, since it entails observations of discursive instances through extensive discourse analysis. Therefore, a practical heuristic method is to model the description of one language on the description of another. This is the method of transfer comparison [38, 39]. Fundamentally, transfer comparison draws attention to resemblances between two languages [39]. We described the system network for the current annotation scheme by using the method of transfer comparison, making descriptive assumptions based on English, since although the system network for English has already been delineated in previous studies [37, 40], one for the Japanese language had not been systematized yet.

**Fig 5:**
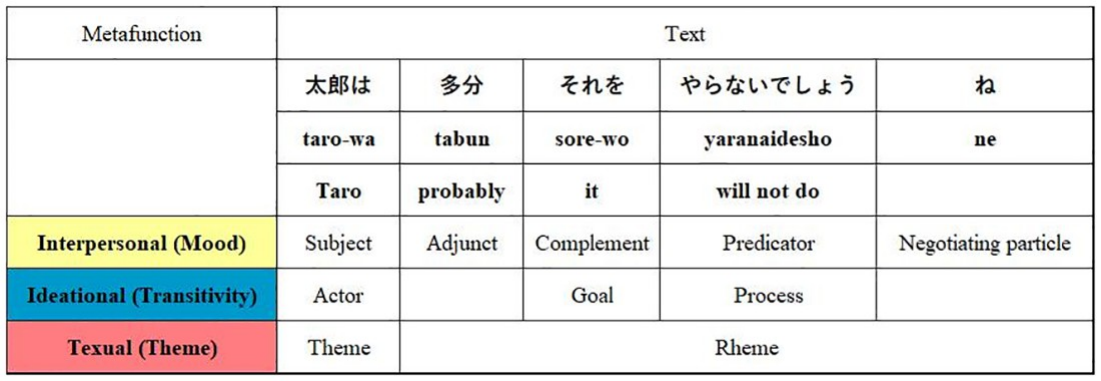
The relation between metafunctions and a text. Text denotes every interaction, whether spoken or written, unfolding in some context of use [34].

The description is made by classifying items of lexicogrammar from the functional point of view, categorizing them into one of the three meta-functions, ideational (which functions to construe external and internal reality), interpersonal (which functions to enact social relationships), and textual (which functions to create text of semiotic reality), which SFL has set up. In linguistic activity, lexicogrammatical choices are made from the system network for each of these three metafunctions. Metafunction does not constitute a paradigmatic appositional structure in which one metafunction is assigned to one clause, but an integrated structure in which one clause is composed of three metafunctions, as shown in Fig 5. In other words, texts are analyzed from the multilayered metafunctional structure, which enables densely-delicate mapping of semantic analysis of texts.

SFL theory is comprehensive, since SFL “is concerned with language in its entirety, so that whatever is said about one aspect is to be understood always with reference to the total picture, as can be seen in the detailed system network. At the same time, of course, what is being said about any one aspect also contributes to the total picture [41].” There is no other language theory that so comprehensively grasps a language as a system. This is why we assume the SFL approach best fits the development of the annotation scheme of the current corpus.

### (3) Creating the dictionaries for annotation

In order to proceed with annotation using the tool developed for this study, dictionaries must be stored in the tool in advance. We created 15 dictionaries for lexicogrammatical annotation along the system network.

### (4) Development of the annotation tool + annotation

We developed the annotation tool, which facilitates the annotation procedure, for this project. Our annotation tool enabled us to establish a streamlined process. Annotation was performed by four annotators: a doctor of linguistics, an MA researcher in Japanese education as a second language, and two others with an MA and a BA each. The lexicogrammatical categories are based on linguistic concepts, but some can accommodate various interpretations; as a result, judgements often fluctuated among annotators. In order to minimize these fluctuations and achieve a high agreement rate, the coding criteria for each category were set as minutely and delicately as possible. However, there often occurred occasions requiring careful consideration, and the final confirmation was negotiated among the four annotators. In addition, owing to a 2% chance of error in the segmentation of morphemes by UniDic-MeCab, morphological analyzer, developed by the National Institute for Japanese Language and Linguistics, further meticulous checking of them was required.

### (5) Designing the corpus

After designing and programming the quantifying, visualization, and search functions, as well as the interface and other factors, the tagged data were stored and the corpus was configured.

### (6) Machine learning of annotation

The technology of natural language processing would give birth to corpora which enable easy access to huge amounts of elaborated information. The disadvantage is that the cost, manpower, and time required to build a corpus are enormous, although commensurate results can be expected. In order to reduce this workload, we pursued the possibility of automation of the annotation by machine learning. This technology is integral to the achievement of group discrimination among subjects, which is our ultimate goal to be carried out in future research.

**Fig 6:**
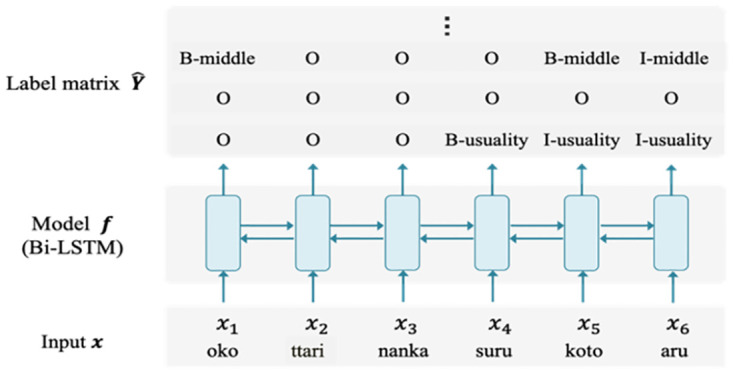
IOB2 notation—Schematic diagram of the tagging prediction process by Bi-LSTM. “*Oko ttari nanka suru koto aru* (There are occasions when I get mad)”. *Middle* denotes a type of verb form with a perspective lacking agency. *Usuality* is a type of modality expressing how often an event tends to occur. Further explanation is provided in Table 4. Both are selective resources embedded in the system network.

We formalized the annotation process as a sequence labeling problem of predicting tags for words in a sentence [42]. IOB2 notation was applied to each tagging [43]. IOB2 is a notational system that assigns the label O to untagged words, B- (tag name) for the first word of the tagged span, and I- (tag name) when the same tag name appears in the rest of a span, as shown in Fig 6. The IOB tags in Fig 6 indicate that value *middle* is assigned to *oko* and *koto aru* and value *usuality* to *suru koto aru* in the sentence “*Oko ttari nanka suru koto aru* (There are occasions when I get mad)”. The procedure is to train a machine to learn which of the labels should be assigned to each word.

This sequence labeling is performed using a deep neural network (DNN). A DNN is a kind of machine learning model. Giving it a large number of input and output pairs (examples) enables the machine to learn complex relationships between elements. This method has been applied in various fields such as image recognition [44, 45] and automatic translation [46, 47], and has achieved great results. While it has very high predictive performance, internal calculations are too complicated for the current level of human-readable technology to explain what and how the machine learns. Hence, it should be kept in mind that there will always be some cases where human judgement is required.

An overview of tag prediction by the DNN model is shown in Fig 6. The DNN model ƒ has parameter θ and calculates the label matrix $\hat{\text{Y}}$ for each word of the sentence consisting of T words. That is, $\hat{Y}=f\left(x;\theta \right)$. Here, $\hat{\text{Y}}$ is a C × T matrix where each row represents a tagging type and each column represents a word. The parameter θ to perform the calculation in an appropriate manner is learned using training data, which is an example to teach the model the relationship between x and Y. More specifically, it is a set of pairs of input x and output Y. The learning of Parameter θ is formulated as an optimization problem to minimize the loss between the prediction $\hat{\text{Y}}$ by model ƒ and the correct answer Y.

The sequence labeling was performed by a one-layer Bi-LSTM [48], which is a standard DNN model used in the field of NLP as a better model than unidirectional LSTM. The main advantage of Bi-LSTM over unidirectional LSTM is that the latter cannot theoretically consider words spoken later in a sentence after a particular target word is spoken. For example, when estimating the tag of “B” in the word sequence “A B C”, it may be important that “C” follows. In this case, Bi-LSTM can take it into account, but unidirectional LSTM cannot.

We trained the model for 50 epochs with a batch size of 32 using the Adam optimizer with a learning rate of 0.001 [49]. The dimension of the input word vectors and the hidden layer was 300. All parameters, including word embeddings, were randomly initialized.

### (7) Exemplifying PI mapping

We exemplified the procedure of mapping lexicogrammar considered to indicate PI, taking negotiating particles as an example. Negotiating particles are sentence-ending particles in the Japanese language. We examined the different choices of negotiating particles made by two groups in Task A (interview) targeting late adolescents and adults with ASD (n = 50) and TD counterparts (n = 57), whose age had exceeded the critical language learning period. We conducted t-test to investigate the difference in the occurrence rate of the negotiating particles in the two groups.

## Results

### (1) The construction of system networks

**Fig 7:**
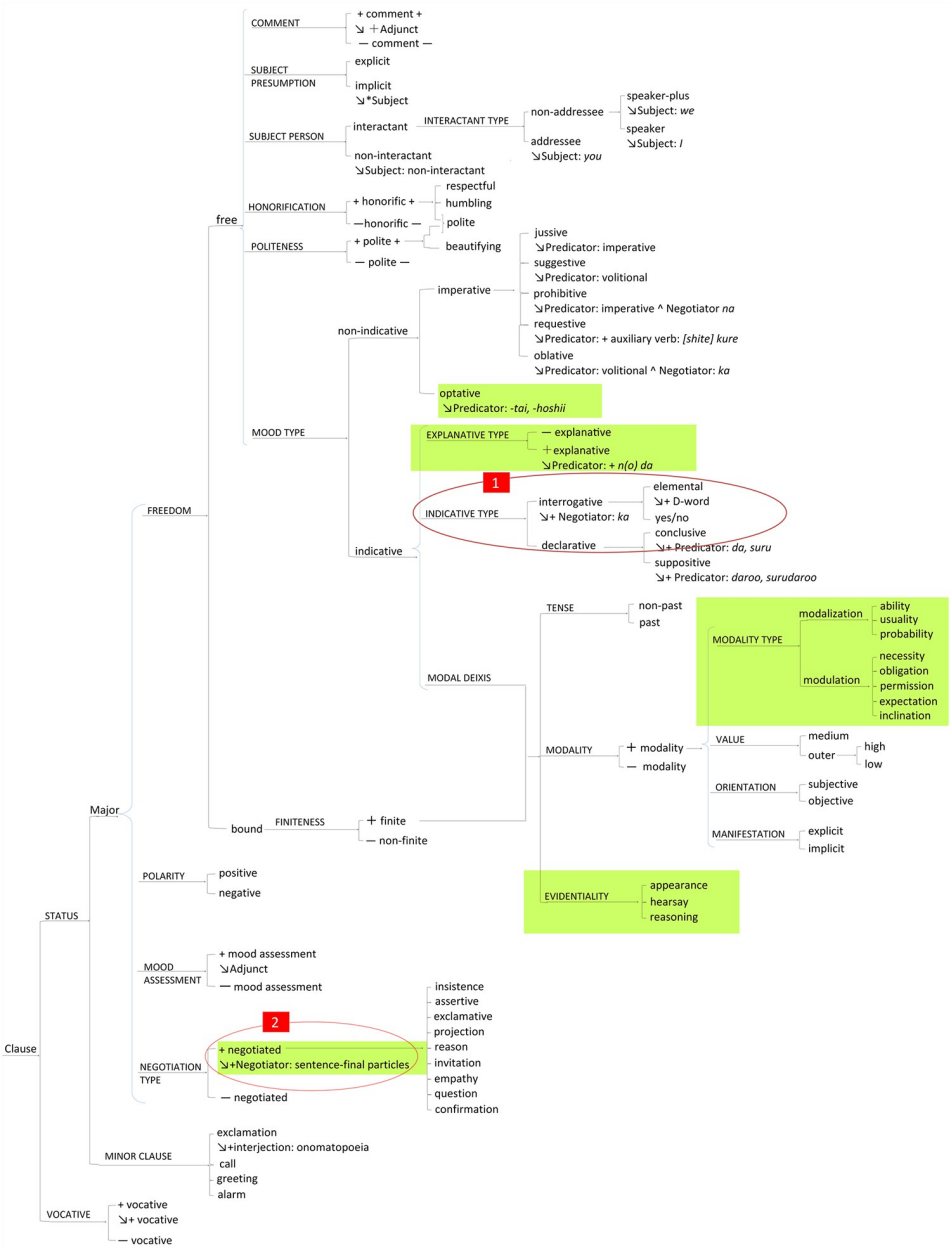
The system network of MOOD (mood selection) in Japanese from the interpersonal metafunction. This was modified from Teruya [50] and Kadooka, Kato, Iimura et al. [51]. The red-circled Part 1 is in Fig 4, and the red-circled Part 2 is discussed as Identifying PI in negotiating particles in Results. The speakers are expected to choose one from a set of oppositions, with the degree of delicacy increasing from left to right on the network. The current annotation scheme incorporated the items in the green-colored portions.

**Fig 8:**
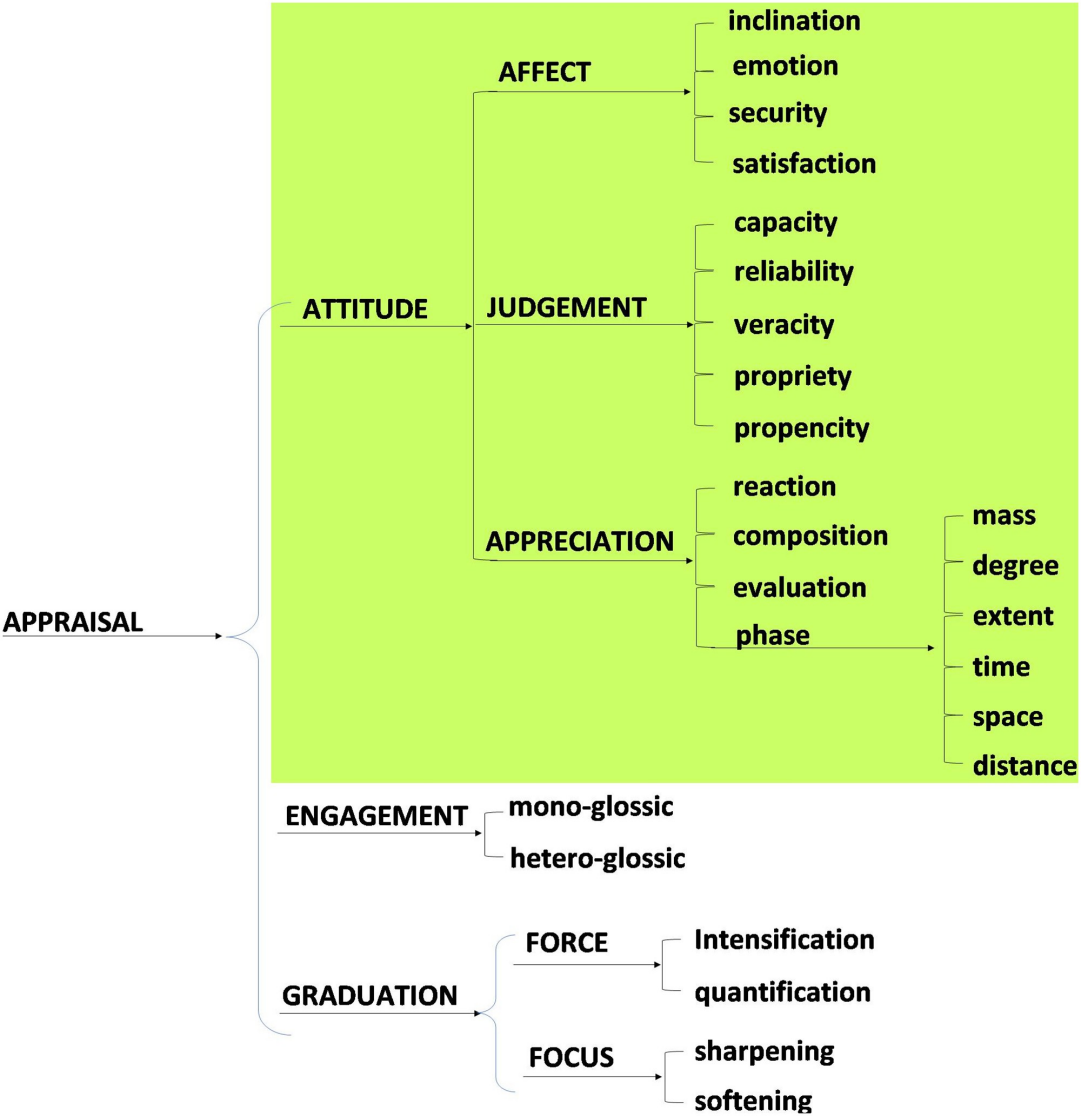
System network of APPRAISAL in Japanese from the interpersonal metafunction. This was constructed by transfer comparison following Martin and White [52]. The current annotation scheme incorporated the items in the green-colored portions.

**Fig 9:**
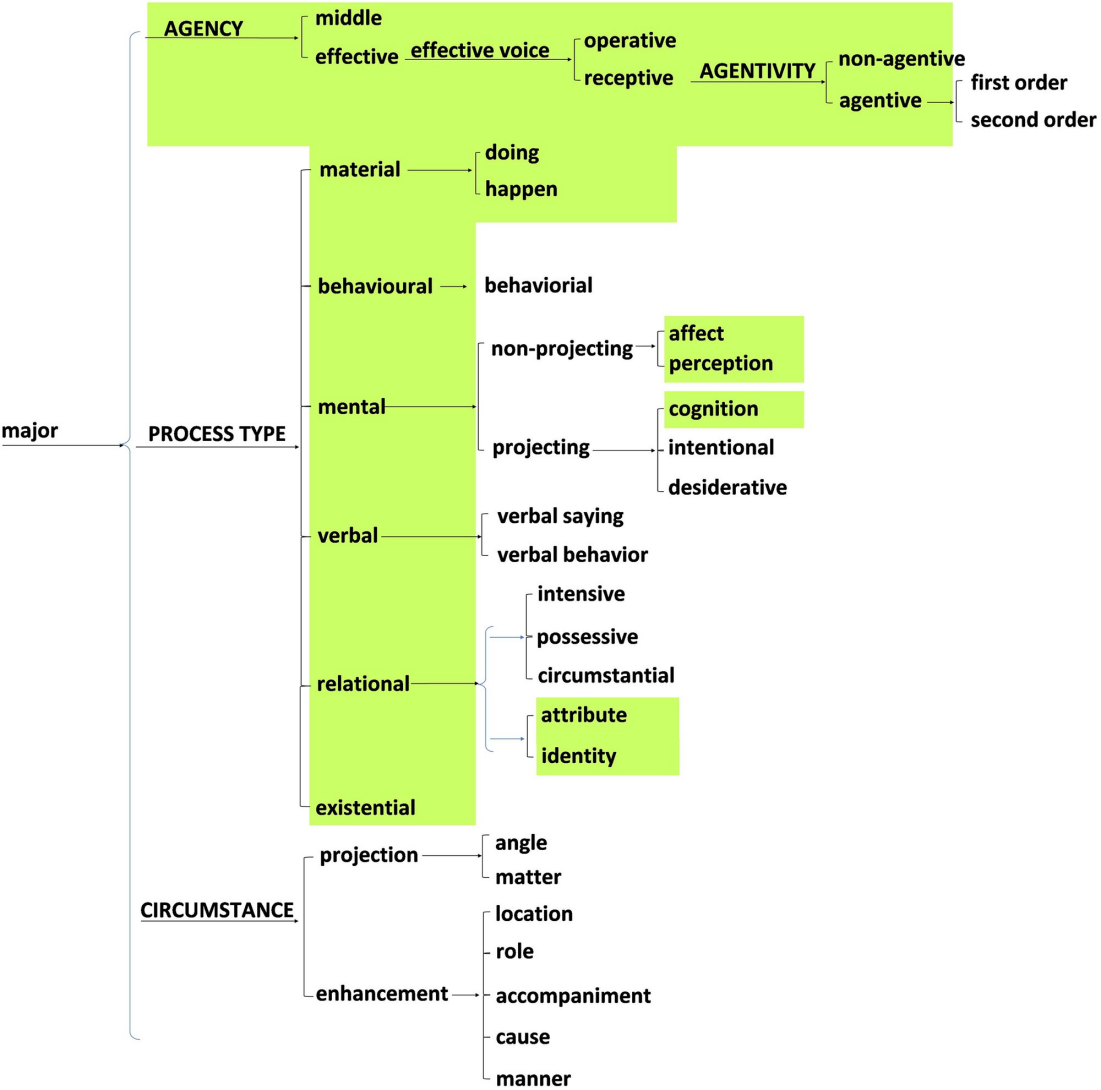
System network of TRANSITIVITY in Japanese from the ideational metafunction. This was constructed by transfer comparison following Matthiessen [40]. The current annotation scheme incorporated the items in the green-colored portions.

**Fig 10:**
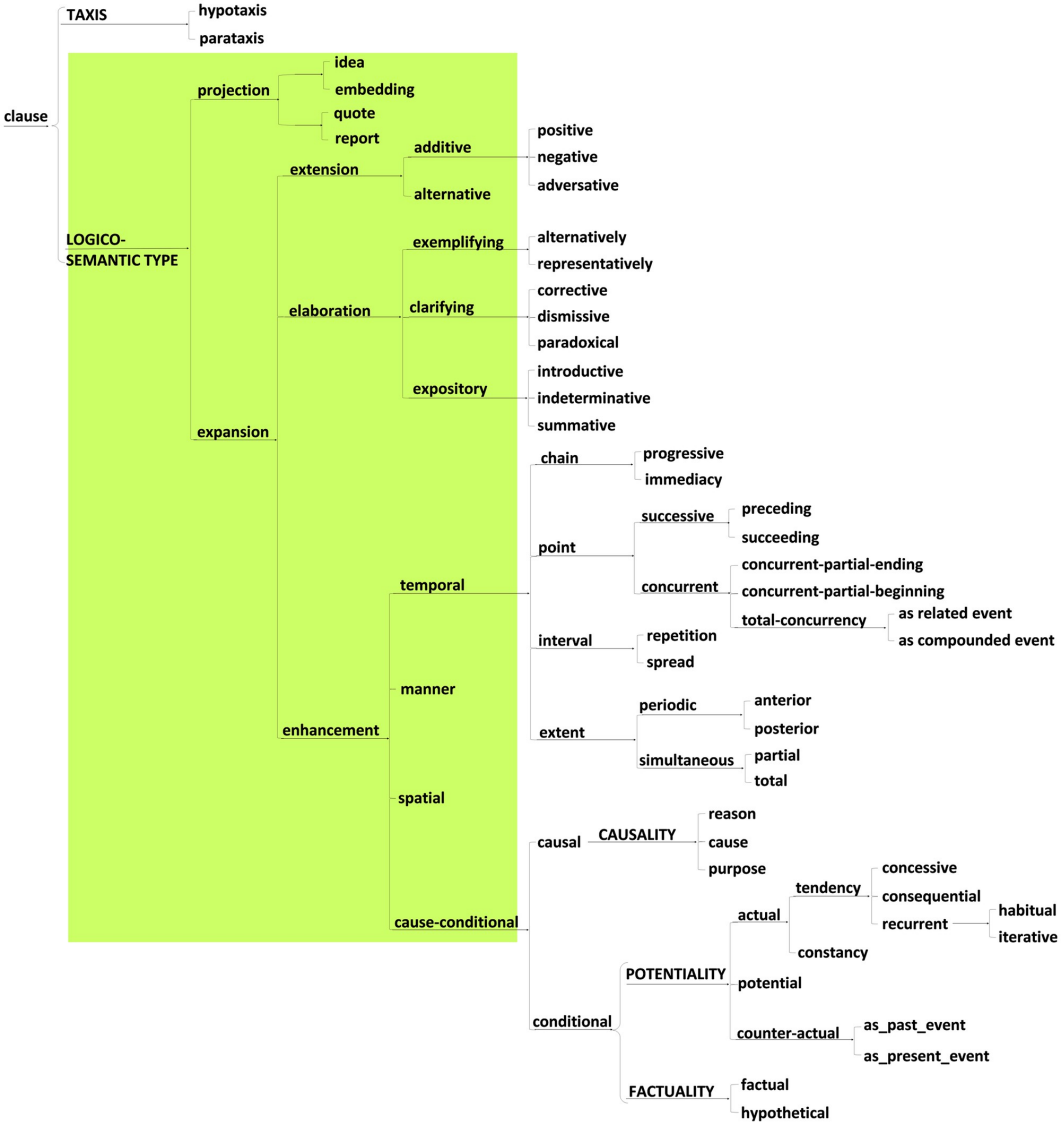
LOGICAL systems in Japanese from the ideational metafunction. This was constructed by transfer comparison following Halliday [41]. The current annotation scheme incorporated the items in the green-colored portions.

Using the method of transfer comparison with English, we have constructed the four system networks that are included in two of the metafunctions in the Japanese language. These four are (1) the system of MOOD in Japanese (Fig 7), and (2) the system network of APPRAISAL in Japanese, both from the interpersonal metafunction (Fig 8). Then, (3), the system network of TRANSITIVITY in Japanese (Fig 9) as well as (4) the LOGICAL systems in Japanese (Fig 10) arise from the ideational metafunction. We systemically mapped all of the potential lexicogrammatical choices from among all of the categories of grammar and semantics delineated throughout Figs 7–10. We mapped the possible language constructs for these four systems (mood, appraisal, transitivity, and logic) as a network of interlocking options while designing the annotation scheme in the network for the current corpus, which does not cover all the lexicogrammar in the system network, but does the green-colored sections, Figs 7–10. In construction of the corpus, the recommended use is to select the resources needed to incorporate items of lexicogrammar into the annotation scheme depending on what the research intends to investigate or to elucidate. Although we have now identified and then incorporated a very wide range of resources in the corpus, the corpus will necessitate and accommodate even further resource expansion as we and other researchers come to use it to aid in the investigation of particular new areas. Lexis and items of lexicogrammar pertaining to each particular application of research can flexibly be built into the corpus.

### (2) Development of the annotation scheme

Table 3 shows the scheme of tagset and the lexicogrammatical information which is derived from the system networks for annotation. Each heading has its own subcategories, resulting in a total of 159 items. It can be assumed that individuals with ASD hardly ever choose correctly among these 159 items, due to features of their central cognitive dysfunction in the areas of executive function, joint attention, and central coherence. They exhibit some deficiency in the cognitive ability that seems necessary to use appropriate items of lexicogrammar functionally.

**Table 3:** Headings of the semantic annotation scheme.

headings of annotated lexicogrammar	tag types	number of tag types
**Ideational metafunction**		
1. Process type	1.Material-doing 2.Material-happen 3.Mental-cognition 4.Mental-affect 5.Mental-perception 6.Relational-attribute 7.Relational-identity 8.Behavioral 9.Verbal 10.Existential	10
2. Ergativity	1.effective 2.middle	2
3. Transitivity	voice (1.passive/active 2.causative)	2
4. Clause complexes	1.Parallel clause 2.Te-form/Conjunctive clause-parallel/contrast 3.Te-form/Conjunctive clause- forerunner 4.Te-form/Conjunctive clause-sequence of actions 5.Te-form/Conjunctive clause-cause/ reason 6.Te-form/Conjunctive clause-adversative connective 7.Te-form/Conjunctive clause-resultative condition 8.Te-form/Conjunctive clause-attendant circumstance 9.Conditional clause-resultative condition 10.Conditional clause-converse condition-converse condition 11.Conditional clause-converse condition-adversative connective 12.Conditional clause-cause/ reason 13.Purpose clause 14.Time clause-temporal anteroposterior relation 15.Time clause-simultaneous actions 16.Time clause-others 17.Manner clause 18.Reported clause 19.Interrogative clause 20.Noun clause 21.Adnominal clause 22.Cordinate clause	22
5. Logico-semantic relation	1.Expansion-elaboration-expository 2.Expansion-elaboration-exemplifying 3.Expansion-elaboration-clarifying 4.Expansion-extension-additive 5.Expansion-extension-alternative 6.Expansion-enhancement-temporal 7.Expansion-enhancement-spatial 8.Expansion-enhancement-manner 9.Expansion-enhancement-cause-conditional 10.Projection-quote 11.Projection-report 12.Projection-idea 13.Projection-embedding	13
6. Auxiliary verbs	stative: (19 categories) compound: (13 categories)	32
**Interpersonal metafunction**		
7. Modality	1.Ability 2.Probability 3.Usuality 4.Necessity 5.Obligation 6.Permission 7.Expectation 8.Inclination	8
8. Appraisal: Attitude	1.AFFECT-inclination 2.AFFECT-emotion 3.AFFECT-security 4.AFFECT-satisfaction 5.JUDGEMENT-capacity 6. JUDGEMENT-reliability 7.JUDGEMENT-veracity 8.JUDGEMENT-propriety 9.JUDGEMENT-propencity 10.APPRECIATION-reaction 11.APPRECIATION-composition 12.APPRECIATION-phase-time 13.APPRECIATION-phase-extent 14.APPRECIATION-phase-degree 15.APPRECIATION-phase-space 16.APPRECIATION-phase-distance 17.APPRECIATION-phase-mass 18.APPRECIATION-social evaluation	18
9. Appraisal: Graduation	1.FORCE-intensification 2.FORCE-quantification 3.FOCUS-sharpening 4.FOCUS-softening	4
10. Negotiating particle	sentence-ending particles; 1.*kana* 2.*kane* 3.*sa* 4.*ne* 5.*yo* 6.*yona* 7.*yone*: Particle- 8.*kane* 9.*sa* 10.*ne* 11.*yo*- at places other than the end of the sentence: other- 12.*ne*	12
11. Explanative mood	1.Explanative mood 2.Explanative mood-*ka* 3.Explanative mood-*kana* 4.Explanative mood-*kane* 5.Explanative mood-*kedo* 6.Explanative mood-other 7.Explanative mood-*na* 8.Explanative mood-*ne* 9.Explanative mood-*yo* 10.Explanative mood-*yone* 11.Explanative mood-*yona* 12.Explanative mood-*monoda*	12
12. Evidentiality	1.hearsay 2.reasoning 3.appearance	3
13. Optative mood	lexis to express desire to do something	1
6. Auxiliary verbs	Benefactive: (10 categories)	10
14. Onomatopoeia	1.imitative word 2.imitative mimetic word	2
15. Filler	Filler words-1.*maa* 2.*nanka* 3.*ano* 4.*unto* 5.*eeto* 6.*sono* 7.*kono* 8.*kou*	8
	total	159

We created 15 dictionaries that collect lexis corresponding to these headings shown in Table 3. The categories in the dictionaries are process type, ergativity, voice, auxiliary verbs, clause complexes, logico-semantic relation for ideational metafunction, modality, evidentiality, attitude, graduation, negotiating particle, mood, onomatopoeia, and filler for interpersonal metafunction, with 159 different tag types in all.

The ways in which speakers use each item of lexicogrammar provide valuable clues that help researchers understand PI of ASD, as noted in Table 4.

**Table 4:** Linguistic functions of annotated lexicogrammar.

headings of lexicogrammar	Linguistic Functions
**ideational metafunction**	
Process type	The mental image of reality is constructed by the clause component, TRANSITIVITY, which allows us to create a representation of reality. We are able to define our experiential world through 10 types of process verbs, Material-doing, Material-happen, Mental-cognition, Mental-affect, Mental-perception, Relational-attribute, Relational-identity, Behavioral, Verbal, and Existential. This lexicogrammar provides information of how the speaker tends to create a representation of reality.
Ergativity	The ERGATIVE system is modelled by causation or instigation. In an ergative analysis, the participant that causes an event is referred to as the *agent*. Ergativity provides information about whether the speaker construes events and reality from the causal viewpoint of *agency* (ergative-effective) or *becoming* (a perspective without agency called ergative-middle).
Transitivity	Transitivity provides clues about the perspective, active or passive, from which the speaker construes events and reality.
Clause complexes	Observation of which of the 22 Japanese sentence types the speaker chooses will reveal the speaker’s syntactic ability and cognitive tendency or deficiency.
Logico- semantic relation (LSR)	LSR concerns how clauses are linked to one another logically, revealing the speaker’s syntactic ability, discourse strategy, and cognitive tendency or deficiency.
Auxiliary verbs	**Stative**: These are a group of verbs describing a state of the subject rather than action; as such, they reflect the speaker’s perspective on the ongoing phenomenon.
	**Compound**: These are a group of verbs created by adding one verb to another verb’s stem; they reflect the speaker’s morphological skill.
**interpersonal metafunction**	
Modality	In SFL, modality refers to the area of meaning that lies between yes and no–the intermediate ground between positive and negative polarity, being categorized as two types, modalization and modulation. While modalization (equivalent to epistemic modality) represents the speaker’s appraisal of ability, probability/predictability, and usuality, modulation (equivalent to deontic modality), represents necessity, obligation, permission, expectation, and inclination.
Appraisal- attitude	Evaluative lexis setting up the semantic resource to negotiate emotional reactions, judgements of behavior, and valuation of things. Attitude is divided into three domains: affect, judgement and appreciation. Affect is the resource used for construing emotional responses (fear, loathing, sadness, happiness, etc.), judgement is for moral evaluations of behavior (ethical, brave, deceptive, etc.), and appreciation construes the ‘aesthetic’ qualities of semiotic phrases/processes and natural phenomena (remarkable, desirable, elegant, harmonious, innovative, etc.). This lexicogrammar reveals the speaker’s value system.
Appraisal- graduation	Along with attitude, graduation is one of the three evaluating domains, concerning gradability, which works for adjusting the degree of an evaluation. Graduation sets up two axes of gradability–force and focus, each of which has two sub-categories, intensification/quantification for force, and sharpening/softening for focus.
Negotiating particle	Negotiating particle is lexis to add various negotiatory values to the clause, which implies the speaker’s attitudinal stance towards the proposition or proposal, concerning call-for attention and territory of information.
Explanative mood	Explanatory mood is an optional type of lexicogrammar often added to the other mood types such as declarative and interrogative, implying a variety of meanings; e.g. cause, reason, motivation, source and grounds for judgement implying a causal relationship between the explained and the explainer.
Evidentiality	Evidentiality is how the speaker’s judgement is made in regards to the validity of the proposition [50]. Three types of evidence as a judgemental standard are appearance, source, and reasoning. Appearance refers to ‘how the information is likely to appear or eventuate,’ source to how it comes to be known to occur, and reasoning to ‘for what reason it is judged or known to happen’.
Optative mood	The optative mood refers a ‘desire’ or ‘urge’ to do something that is regarded as desirable from the speaker’s point of view.
Auxiliary verbs, benefactive	Benefactive auxiliary verbs are verbs used between two parties, one doing something for someone’s benefit and the other being the benefit recipient, reflecting whether the speaker positions the other party inside or outside.
Onomatopoeia	There are two kinds of words, imitative and mimetic, to be used to express manner, quality, exclamation, etc.
Filler	Filler is a time-filler in the form of meaningless sound, word, or phrase in social settings where they are aware of the listener’s presence.

### (3) Specifications of the annotation tool we developed

The annotation tool we developed consists of four different parts, List, Edit, Search, and Corpus. The procedure is that the tool first morphologically analyzes sentences in the corpus using the UniDic-MeCab morphological analyzer. There are various levels of annotations, and part-of-speech (POS) tags are among general annotations. POS tags are the most basic tagging made according to morphological analysis, and tools for automatic tagging have been developed. Owing to the nature of the Japanese writing system, in which character strings are connected seamlessly (without word breaks), although the percentage is slight, there is usually a degree of fluctuation in the classification of POS. The current corpus adopts UniDic-Mecab as the morphological analyzer. Then it searches for matches with lexicogrammar in the dictionary. The discovered lexicogrammar can be verified manually by clicking the check button. In addition, the tool can add new lexicogrammatical items to the dictionary based on the surface form, basic form, and part-of-speech of words, by applying the principles of the algorithm. This tool has been implemented as a web application adopting Python as the backend. The 15 dictionaries described in Subsection (2) above were built into this annotation tool.

### (4) The structure of the corpus

**Fig 11:**
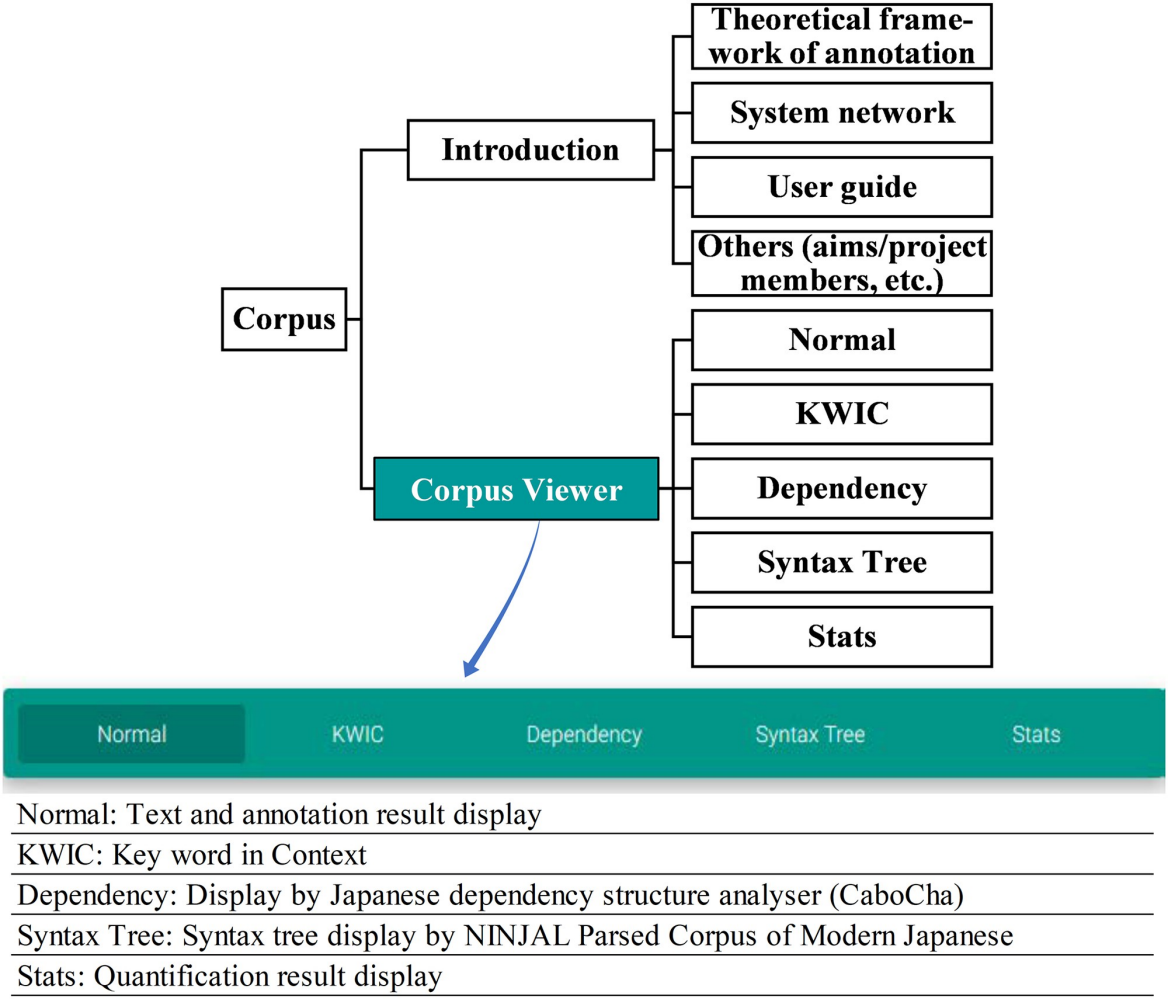
The structure of the corpus. The corpus is divided into two main parts, the Introduction and the Corpus Viewer. The Corpus Viewer incorporates the search and results display screen.

We designed the corpus structure as shown in Fig 11. The completed corpus has two main parts, the Introduction and the Corpus Viewer. In the Introduction, the theoretical framework and system network related to the annotated lexicogrammar are included. Data retrieval is performed in Corpus Viewer, of which the central function screens are Normal, KWIC (Key Word in Context), and Stats. The detailed Corpus Viewer with description is provided in S1 File.

The current corpus has two other screens, CaboCha and Tree View, both of which are publicly released. CaboCha is a Japanese dependency structure analyzer, and Tree View is from the Parsed Corpus of Modern Japanese published by NINJAL (National Institute for Japanese Language and Linguistics) [53].

In summary, as to what can be understood from the current corpus, the frequency of the lexicogrammar used is instantly displayed graphically, allowing comparison among the groups of interview subjects. High frequency is regarded as an index of generality, standardization, and typicality of lexicogrammatical use. The frequency obtained can be employed for statistical investigation in order to map the lexicogrammatical usage, for the purpose of identifying where PI occurs in the system network.

### (5) Machine learning of annotation: Toward automation of semantic tagging

In order to evaluate the performance of automatic tagging, we divided the manually verified data into training, development, and test sets by the ratio of 8:1:1. The machine learning model was trained on the training data, and the hyperparameters were tuned on the development set. We then measured the performance of the trained model on a test set.

Precision, recall, and F1 are used as the evaluation metric. Precision is the percentage of tags predicted by the model that were correct, recall is the percentage of tags that the model could predict among the gold tags (that is, the tags that should be predicted), and F1 is their harmonic mean. That is:

$$Precision=\frac{Number\;of\;correctly\;predicted\;tags}{Number\;of\;tags\;output\;by\;the\;model},$$

$$Recall=\frac{Number\;of\;correctly\;predicted\;tags}{Number\;of\;human-annotated\;tags},$$

$$F1=\frac{2Precision\cdot Recall}{Precision+Recall}$$

For example, suppose it is correct to assign *middle* to *oko* and *koto aru*, and *usuality* to *suru koto aru*, as shown in Fig 6. Also, suppose that the model predicts that *middle* is assigned to *oko* and *suru*, and *usuality* to *oko* and *suru koto aru*. Then, precision, recall, and F1 are 0.5 = 2/4, 0.66 = 2/3, and 0.57, respectively.

F1, precision, and recall on the test set were 0.88, 0.89, and 0.87, respectively. This result is regarded as reliable enough to be utilized for group discrimination at a later stage. As far as reduction of workload is concerned, automation of annotation reduces the amount of work considerably whenever data are added and the corpus is expanded in size.

#### Identifying PI in negotiating particles, and constructing the model flow of pragmatics

We are currently proceeding with the mapping of PI and cognitive reasoning targeting all the annotated lexicogrammar. We will exemplify the procedure of mapping lexicogrammar considered to be PI by taking as an example the negotiating particles, sentence-ending particles in the Japanese language, which are included in the interpersonal metafunction from the annotation scheme (see the red-circled Part 2 in Fig 7). We conducted t-test to investigate the different choices of negotiating particles made by two groups, late adolescent and adult ASD individuals (n = 50) and their TD counterparts (n = 57) in Task A (interview). Table 5 shows the results of the Welch’s t-test, indicating there were significant differences (p < .05) in choices of three negotiating particles, *kane* (p = .025), *ne* (p = .003), and *yo* (p = .008) between the two groups.

**Table 5:** Results of Welch’s t-test.

	Mean in TD	STD in TD	Mean in ASD	SD in ASD	t	p
*kana*	0.003071	0.001896	0.003423	0.003519	-0.623	0.535
*kane*	0.002423	0.002964	0.001179	0.002630	2.267	0.025
*sa*	0.000000	0.000000	0.000017	0.000094	-1.276	0.208
*ne*	0.010057	0.008505	0.005572	0.006585	3.024	0.003
*yo*	0.001900	0.002430	0.000839	0.001467	2.729	0.008
*yona*	0.000045	0.000126	0.000026	0.000130	0.757	0.451

SD represents standard deviation.

Thus, less use of *ne*, *yo*, and *yone* in comparison with TD, is regarded as PI. *N*e and *yo* concern calls for attention measuring the ownership of information concerned. These two negotiating particles are used differently depending on how the speaker gauges ownership of the related information [54]. Maynard’s analysis including *yo* is summarized in Table 6. Although *yo* is the particle that focuses on the information itself, there are cases where the simple *yo* is avoided, even by speakers who possess more information, i.e., when S’s (Speaker’s) amount of information is greater than H’s (Hearer’s). In such cases, the *yone* combination may be preferred, as shown in the middle row of the Table 6 [54].

**Table 6:** The use of the negotiating particles *ne* and *yo* according to possession of information by speaker and hearer [5,4].

the relative degree of possession of the information	particle chosen
S exclusively holds the information; H does not have any	*yo*
H exclusively holds the information; S does not have any	*ne*
S’s amount of information > H’s amount of information	*yo or yone*
H’s amount of information > S’s amount of information	*ne*
S’s amount of information = H’s amount of information	*ne*

Pragmatic functions of *ne* from the calling-attention perspective are: (1) When the speaker intends to unfold the interaction, s/he firstly brings up the topic and then involves the hearer by using *ne*, (2) When the speaker desires conversational bonding with the hearer, *ne* indicates that the speaker is going to share the topic and information given by the hearer, and (3) When the topic or information is already shared, the speaker seeks the hearer’s agreement or confirmation by using *ne* [55].

Particles play an indispensable role in building interpersonal relationships, among which *ne* and *yo* are of central importance in the Japanese socio-cultural context. *Ne* is the principal expression for placing the hearer in the position of a fellow interlocutor, while *yo* tends to forcefully push the speaker’s idea or judgement toward the hearer [56]. From the viewpoint of old/new information, *ne* functions interpersonally by indicating that an ongoing utterance constitutes a shared, two-person matter. On the other hand, *yo* draws attention to the content of what is being said by indicating that the speaker’s words are delivering new information [57]. Briefly, the two negotiating particles, *ne* and *yo*, both of which are frequently used in spoken Japanese, are chosen for use in the system network based on the pragmatic rule that *yo* or *ne* is selected only after judging whether or not the hearer already has the information concerned. Comparing these two negotiating particles, *yo* is functionally stronger than *ne* in calling for attention.

Thus call-for-attention is the central pragmatic function of *ne*, *yo*, and *yone*. Call-for-attention entails JOINT ATTENTION from the neurocognitive perspective: the negotiating particles *ne* and *yo* can be regarded as non-visual joint attention. The measurement of the ownership of information, which is one of the factors governing the use of *ne*, is mainly associated with central coherence. The use of *ne*, *yo*, and the combined form *yone* requires the neurodevelopment of a global processing ability to integrate such information as the hearer’s gestures, facial expressions, and other non-linguistic feedback necessary to make appropriate choices from the system network. Such global processing ability is lacking or underdeveloped in individuals with ASD. Collaterally, such early social information processing impairment leads to subsequent impairment in the development of social knowledge and social cognitive skills [58]. Consequently, individuals with ASD generally continue to suffer from not necessarily unimprovable but still unconquerable social interaction problems in adulthood.

The premise of this research is that each feature of lexicogrammar has a function, and that each function, in turn, is associated with a certain neurocognition that is required to use it. Therefore, the inability to use a given lexicogrammatical element properly in a social situation means that its associated neurocognition is flawed (i.e., defective).

**Fig 12:**
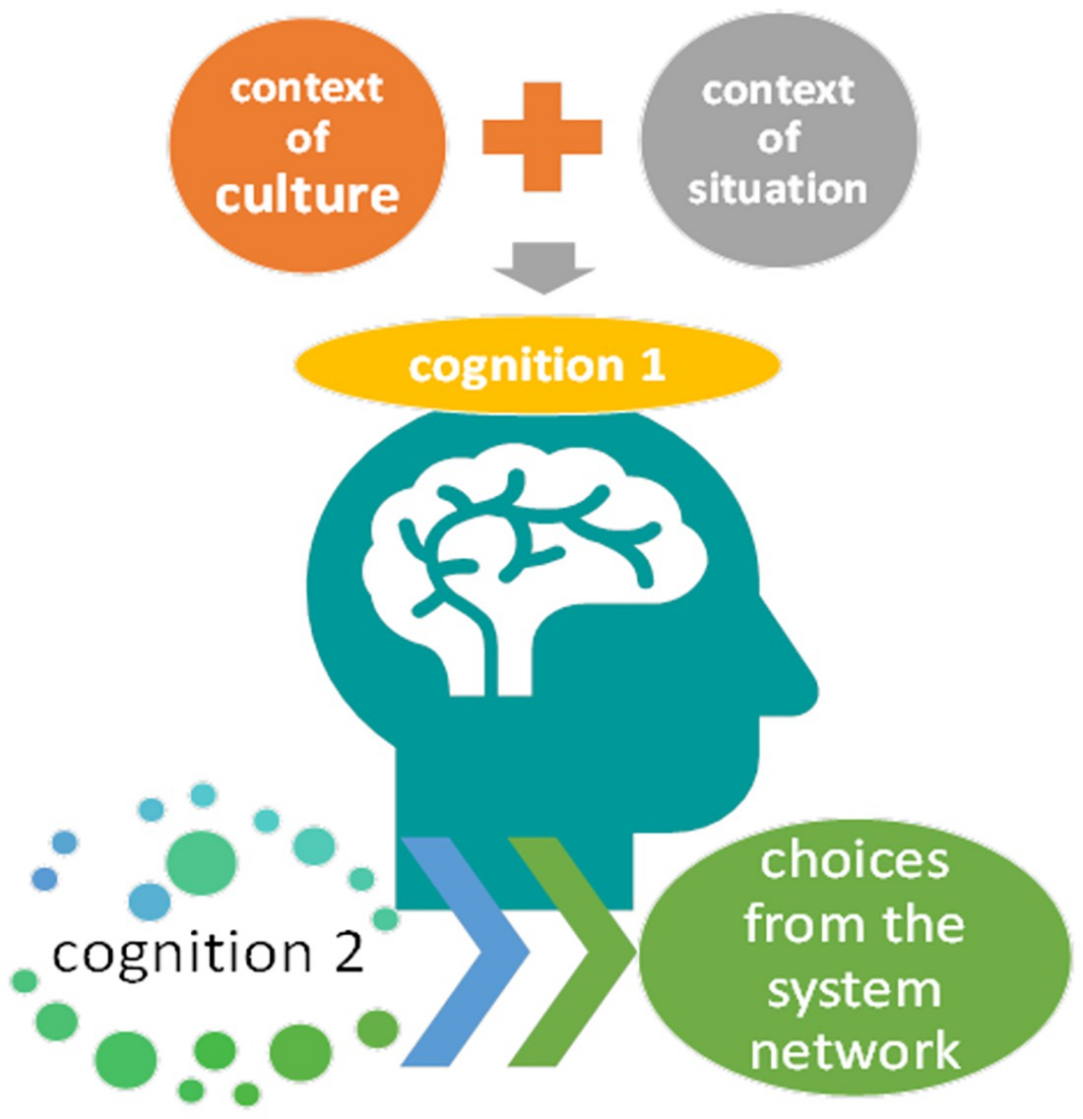
Model flow of pragmatics defined in the theoretical framework of SFL.

With all these findings and assumptions taken into consideration, we created the model flow of pragmatics hypothesized for this study as shown in Fig 12. First, the context of culture/situation is recognized or read, and based on that recognition choices are made from the system network. PI involves either or both of two impaired cognitions, cognition 1 and 2. Cognition 1 is associated with reading (or misreading) the two contexts, culture and situation. Cognition 2 is associated with the ability (or inability) to select the specific lexicogrammar that functions most advantageously in a given context. The problem is that individuals with ASD cannot properly recognize the context of culture and situation due to neurocognitive impairment. As a result, a choice different from the TD individual’s choice is made (including the case where no language at all is chosen), which is considered inappropriate for ongoing social interaction. This denotes PI. The current study assumes that the inappropriate utterances of ASD individuals result from deviant choices from the system network [59, 60].

## Discussion

We constructed an annotation scheme for mapping lexicogrammatical PI, based on which we successfully constructed a corpus. Furthermore, by inventing an efficient annotation tool utilizing algorithms and by making a machine learn the annotation, we achieved a precision rate of 90% in automatizing the annotation process. This will enable cost-effectiveness in the expansion of the corpus to the size needed to increase the credibility of findings.

In addition, this study successfully took up a wide examination of the use of negotiating particles by ASD individuals, which are items of lexicogrammar peculiar to the Japanese language, in order to exemplify the mapping process of PI, hypothesizing a relationship between language use and cognitive function. Since choices in the system network are considered as paradigmatic relations or interrelated sets of options, tracing reasons why one particular choice was made, and not others contributes to the mapping of PI and sheds light on the neurocognitive reasoning involved. In other words, consideration of the linguistic function of the lexicogrammar that an ASD speaker selected enables researchers or clinicians to inductively reason which neurocognitive abilities are impaired. This provides insights from the neurocognitive perspective as to why a given choice was made. Thus the primary dysfunction can be inferred from the secondary dysfunction shown in Fig 2. In this way, we use t-test to identify items that have a significant difference between TDs and individuals with ASD in their use of lexicogrammar annotated in the current corpus, collating linguistic functions with cognitive abilities, which finally will end in comprehensive mapping of PI.

### Comparison with prior work

To the best of our knowledge, the corpus constructed by Sakishita et al. [31] is the only other corpus of Japanese-speaking ASD individuals’ spoken language with information tagging. Although there is a difference in that Sakishita’s provides mainly phonetic information, the current corpus goes beyond it in the following respects: (1) The current corpus’s annotation goes beyond the framework of morphemes, while Sakishita’s stays within the morpheme frame, which technically does not transcend the rudimentary level of annotation. (2) The elaborateness and the precision of the current corpus, which has 159 kinds of SFL-based lexicogrammatical annotations, is far beyond the 17 kinds in Sakishita et al. (3) The number of Sakishita’s subjects is 43, with 740 minutes of total interaction time, while the number of subjects in the current corpus is 292 with 1,187 task samples, and although the total time is not calculated, the number of morphemes spoken in the recordings thus far (as tabulated by UniDic-MeCab) is about 1.07 million. Thus the current corpus far surpasses the size of Sakishita’s, which he once called “the world’s largest corpus of Japanese speakers of ASD” [31].

While Sakishita’s is not open to the public, the current corpus is intended to be open to researchers for the purpose of being widely used for the study of PI.

### Contributing points to the field

From the viewpoint of mapping PI in ASD, the following six points are specified as contributing to research in the field.

First, the result of PI mapping gives rise to the possibility that ASD can be diagnosed with the help of observation of lexicogrammatical choices, which is our ultimate goal. The existing ADOS-2 is considered the ‘gold standard’ of diagnostic measures in any diagnostic protocol [61, 62]. However, studies have questioned the versatility of ADOS in discriminating ASD from other conditions, since it has difficulty discriminating among them due to overlapping symptoms [63, 64]. This is especially true for adults [65, 66]. These studies advocate multidisciplinary decision-making on final diagnosis employing diagnostic measures developed from multifaceted perspectives. A measure to detect PI peculiar to ASD individuals is a good candidate as one of these diagnostic tools.

Second, the current corpus provides fundamental information for brain function research. Systematic and elaborate mapping of lexicogrammatical choices by speakers with various types of neurodevelopmental disorders and mental disorders, as well as by TD speakers, is indispensable for the experimental design of language research via brain imaging, such as analysis by fMRI [67]. This corpus can help identify which items of lexicogrammar should be targeted for the imaging experiment. SFL, which is the theoretical underpinning of our corpus, views each aspect of a language with reference to its position in the whole system of that language. This comprehensive perspective is useful for experimental design.

Third, the ASD individuals who contributed to the current corpus were mainly late adolescents and adults. In terms of age, they have passed the critical period for language acquisition. The reason for mainly targeting this age group is due to the hypotheses on the critical period for language acquisition, based on maturation constraints [68, 69], in which the maturation mechanism itself, which is the basis of learning, increases in strength for a certain period of time and thereafter decays or disappears. Humans have an age limit beyond which their ability to acquire language decreases. The language-learning maturity point is a critical period for language acquisition which corresponds to the puberty period. Observation of the lexicogrammar used by the ASD population aged 13 and older reveals which items tend to be missing or improperly used. Findings from this observation, in turn, give language therapists better understanding of what should be reinforced during early ages, critically the 3–6 year-old period. In this respect, the current corpus contributes to improved early intervention.

Fourth, access to the current corpus is expected to attract researchers from interdisciplinary fields such as linguistics, sociology, and medicine, increasing the possibility of interdisciplinary research leading to a more multifaceted understanding of PI.

Fifth, studying the linguistic behavior of people with neurodevelopmental disorders such as ASD via a large-scale corpus provides insight into how normal people use language in their social settings. Because of this, benefits will ensue to the development of pragmatic theory in general.

Sixth, the disclosure of databases such as the current corpus may encourage the construction of similar corpora for other languages. Our method of constructing the annotation scheme and our corpus structure will be applicable to any other languages as well. This is because in SFL, the typological description of the “system network” is applied equally to various languages around the world. Generalizations have been proven valid across languages, through descriptive typology [38].

Each language is distinct in the perspectives of its language description, its system network. However, once comprehensive descriptions of particular languages have been established, there occurs the room for typological generalizations across languages [38]. Transfer comparison enables typological generalization. As long as it stays in the domain of typological generalizations, the PI found in speakers are common across languages. What we must not overlook is the PI that is hidden in one language and manifest in another. The diagnostic protocol of ASD in terms of language use ignoring these phenomena is insufficient. These phenomena become manifest through the annotation scheme constructed from the system network, the description of a particular language. Here is the importance of the identification of cross-linguistic differentiation.

With multilanguage corpora, we will find that some aspects of PI may be manifested or hidden depending on the language [59]. There exists no lexicogrammar in English that exactly corresponds to negotiating particles, the sentence-ending particles in the Japanese language. Conversely, reversing the use of first and second person subject pronouns, which is a characteristic of ASD in English, does not become apparent in Japanese discourse, where the omission of the subject of the clause habitually occurs. Some of the manifestations of lexicogrammatical deficits are language-dependent [4].

Wierzbicka points out the existence of Anglocentric ethnocentrism [70], that is, the tendency in linguistic research to assume that results obtained from the English language are universal phenomena, or that even though there are differences depending on the language, they are so small that they can be ignored. However, Wierzbicka argues that since each language reflects the social culture to which it belongs, there are clear cross-linguistic differences, and thus universalizing linguistic phenomena without scrutiny of those differences would be a problem. The same is true when considering the linguistic behavior of ASD. It is highly expected to systematize PI by clarifying cross-cultural pragmatic differences, and this might be done by identifying phenomena in which deviant use is hidden or manifest depending on the language. The construction and publication of databases for other languages such as the current corpus would give impetus to defining PI from a cross-linguistic point of view. It is hoped that our corpus construction would give rise to a field such as the study of cross-linguistic PI.

### Limitations

The lexicogrammar of the textual metafunction, whose central lexicogrammatical resources are theme/rheme structure, information structure, and coherence, which was not targeted in the current corpus, will be an issue for the future. While textual analysis provides useful information for written texts with well-organized discourse structures, it is not so expedient for more loosely structured spoken language. Depending on the task, however, we might need to reconsider.

## Conclusions

The great advantage of the corpus is that word and phrase usage frequency is instantly provided for statistical investigation. Furthermore, it is significant that a certain amount of task-specific spoken text is visually displayed with analytical results in a well-organized configuration by simple operations in the interface. Although the amount of information is enormous, the specifications are technically designed to realize the maximum efficiency of access. Such a corpus, with a systematically multilayered and elaborated annotation scheme grounded in the theoretical framework of SFL, does not exist in previous studies. For these reasons, the current corpus is a significant step forward in the comprehensive mapping of PI.

At present, this corpus has accommodated a total of about 1.07 million morphemes (as calculated by UniDic-MeCab). As a monitor corpus, we will continuously expand it in size and in the elaboration of semantic annotation over time, in order to make the results more reliable. This is because if the frequency of a certain item of lexicogrammar is zero, it cannot be reliably determined whether that lexicogrammar is simply not used in any situation or whether the corpus is so small that it happens to have not yet appeared [71]. Thus the larger the scale of the data, the more reliable the corpus becomes. Our achievement in automatizing annotation, which reduces cost and saves time, facilitates expanding the corpus size.

Our corpus can store texts of spoken language from psychiatric or therapeutic interviews other than those related to ASD. By expanding the dictionary for annotation, it has great potential to handle any type of text. In the case of data from different fields, the dictionary will be augmented to include annotation items relevant to the research focus. Most importantly, our method of constructing the annotation scheme detailed in this study is applicable to any language. The English version of the current corpus is now under development.

## Supporting information

S1 File Detailed Corpus Viewer with description. (DOCX)
