# Correction: Toward mapping pragmatic impairment of autism spectrum disorder individuals through the development of a corpus of spoken Japanese

**DOI:** 10.1371/journal.pone.0321852

**Published:** 2025-04-01

**Authors:** Sumi Kato, Kazuaki Hanawa, Vo Phuong Linh Nguyen, Manabu Saito, Ryuichi Iimura, Kentaro Inui, Kazuhiko Nakamura

The third author’s surname is missing. The correct name is: Vo Phuong Linh Nguyen. The correct citation is: Kato S, Hanawa K, Nguyen VPL, Saito M, Iimura R, Inui K, et al. (2022) Toward mapping pragmatic impairment of autism spectrum disorder individuals through the development of a corpus of spoken Japanese. PLoS ONE 17(2): e0264204. https://doi.org/10.1371/journal.pone.0264204.
