# Correction: Toward mapping pragmatic impairment of autism spectrum disorder individuals through the development of a corpus of spoken Japanese

**DOI:** 10.1371/journal.pone.0333589

**Published:** 2025-09-29

**Authors:** Sumi Kato, Kazuaki Hanawa, Vo Phuong Linh, Manabu Saito, Ryuichi Iimura, Kentaro Inui, Kazuhiko Nakamura

The following information is missing from the Funding statement: This study was also financially supported by the Hirosaki Institute of Neuroscience, Japan. The funders had no role in study design, data collection and analysis, decision to publish, or preparation of the manuscript.
