# Supplementary material for: Toward mapping pragmatic impairment of autism spectrum disorder individuals through the development of a corpus of spoken Japanese
Source: PLoS One. 2022 Feb 25;17(2):e0264204. doi: 10.1371/journal.pone.0264204 (PMC8880787; doi:10.1371/journal.pone.0264204)
Supplement: S1 File — (DOCX) [file pone.0264204.s001.docx]

**S1 File. Detailed Corpus Viewer with description.**

**S1 Fig 1. Normal screen.** Screen left: Search conditions (File (listed by task and subject group), Tag (annotated lexicogrammar is listed), Age, Gender, Position (Interviewer/Subject/Caregiver), IQ (WISC, WPPSI, K-ABC-Ⅱ, WAIS, Tanaka-Bine V), ADOS/comparison score (only for ASD subjects), ADOS/module used (only for ASD subjects), and Classification (autism, autism spectrum, schizophrenia, TD)). Screen right: Search result display with semantic tags attached to the top of the transcript. The tags on the transcript represent the outcome of the semantic analysis based on the system network. Each tag represents: 1.LSR/Expansion-enhancement-temporal 2.Clause Classification/ Continuous clause/Time clause-simultaneous actions 3.Process Type/Material-happening 4.Agency/ Middle 5.LSR/Expansion-extension-additive 6.Process Type/Material-doing 7.Agency/Effective 8.Appraisal/Attitude/APPRECIATION-phase-time 9.Clause Classification/Continuous clause/Te-form/Conjunctive clause-sequence of actions 10.Others/Polarity/POLARITY-positive 11.Process Type/Existential 12.Process Type/Relational-attribute 13.LSR/Projection-embedding 14.Clause Classification/Attributive clause/Adnominal clause 15.Mood/Explanative Mood 16.Appraisal/ Graduation/FORCE-intensification 17.Others/Polarity/POLARITY-negative 18.Appraisal/Attitude/ AFFECT-security 19.Process Type/Mental-affect.

**S1 Fig 2. Speakers’ logos.** Speakers are color coded (yellow:interviewer / green:subject / white:caregiver) and logos are placed in different positions (left:interviewer / right:subject / both sides:caregiver).

**S1 Fig 3. Display of subject’s information.** Clicking the subject ID makes manifest the subject’s gender, age, IQ, ADOS module used, comparison score of ADOS, and diagnostic classification.

**S1 Fig 4. Display of task materials.** Click the icon on the left side of the transcript turn number on the Normal screen and the material used in the corresponding task will be displayed.

**S1 Fig 5. Search for concordance 1.** Enter "scared of" and click ADD, and the result will be displayed on the KWIC screen.

**S1 Fig 6. Search for concordance.** In concordance searching, exact match or partial match can be selected allowing innumerable search terms.

**S1 Fig 7. KWIC screen.** Along with the semantic tag attached to “scared of”, the sentences in which this expression is used are displayed. Each tag represents: 1.Agency/Middle 2.Process Type/Mental-affect 3.Appraisal/Attitude/AFFECT-security 4.Polarity/POLARITY-negative.

**S1 Fig 8. Discourse display window.** Clicking “scared of” makes manifest the discourses before and after this expression.

**S1 Table 1. Fundamental information displayed on the Stats screen.**

| **type of graph** | **notes** |
| --- | --- |
| Sentences (see 1 ) | The total/average number of sentences |
| Words (see 2 ) | The total number of tokens/token types and MLUm (mean length of utterance in morphemes) |
| Tags (see 3 ) | Frequency of tagged lexicogrammar |
| POS (see 4 ) | Frequency of POS (part-of-speech) used |
| Tagged Phrases (see 5 ) | Frequency of tagged phrases |

**S1 Fig 9. Stats screen.** Display of frequency distribution of the annotated lexicogrammar. The quantified fundamental information available on the Stats screen is shown in Table 1. Place the cursor on the bar of each graph to display the distribution. Also, clicking each graph will enlarge the graph as shown in S10 Fig**.**

**S1 Fig 10. Enlargement of Graph 3, showing frequency of tagged lexicogrammar.** The tags represent: 1.Modalization/Ability 2.Modalization/Probability 3.Modalization/Usuality 4.Modulation/ Necessity 5.Modulation/Obligation 6.Modulation/Permission 7.Modulation/Expectation 8.Modulation/ Inclination 9.Modal Adjunct/Probability 10. Modal Adjunct/Usuality.
